# Case Report: Prenatal diagnosis of 10 fetuses with 15q11-q13 duplication and pregnancy outcome in a cohort of Chinese women

**DOI:** 10.3389/fnins.2025.1613797

**Published:** 2025-09-18

**Authors:** Ke Wu, Xiao Li, Hongmei Zhou, Yan Cong

**Affiliations:** ^1^Prenatal Diagnosis Center, Quzhou Maternal and Child Health Care Hospital, Quzhou, China; ^2^Ultrasonic Department, Quzhou Maternal and Child Health Care Hospital, Quzhou, China; ^3^Rehabilitation Department, Yiwu Maternity and Children Hospital (Yiwu Hospital of Children’s Hospital Zhejiang University School of Medicine), Yiwu, China

**Keywords:** 15q11-q13 duplication, intellectual disability, prenatal diagnosis, genetic counseling, retrospective cohort

## Abstract

The chromosome 15q11-q13 duplication (dup15q) is defined as the presence of three or more copies of 15q11.2-q13.1. The features of the chromosome 15q11-q13 duplication syndrome include developmental delay, intellectual disability, hypotonia, facial anomalies, autism spectrum disorder, seizures, and behavioral problems. To date, more than 120 cases of 15q11-q13 duplication have been reported, but studies on clinical information about prenatal diagnosis of 15q11-q13 duplication and attitudes toward fetuses with 15q11-q13 duplication are extremely limited. Herein, we first report a retrospective Chinese cohort with prenatal diagnosis of 15q11-q13 duplication involving ten phenotypically normal pregnant women. In this Chinese cohort, most of fetuses (90%) with 15q11-q13 duplications did not have ultrasound abnormalities. Although the penetrance of 15q11-q13 duplication appeared to be incomplete, 60% of families opted to terminate the pregnancy due to concerns about potential risks. Not all 15q11-q13 duplications were attributed to the parental inheritance, 15q11-q13 duplications could also be caused by parental genomic structure variation. We hope that this study provides new and useful insights into the prenatal diagnosis of 15q11-q13 duplication, thereby informing genetic counseling and decision-making regarding pregnancy outcomes.

## 1 Introduction

The chromosome 15q11-q13 duplication is defined as the presence of three or more copies of 15q11.2-q13.1 and usually has two forms: isodicentric chromosome 15q (idic(15)) and interstitial 15q11-q13 duplication. Individuals with an idic(15) generally exhibit more severe clinical phenotypes compared to those with an interstitial duplication (DiStefano et al., 2016). The phenotype of 15q11-q13 duplication depends on the parent-of-origin of extra copies of 15q11-q13 duplication. Individuals with paternal duplications are generally considered phenotypically normal. In a few cases, they may exhibit milder clinical phenotypes compared to those with maternal duplications (Parijs et al., 2024). Although more than 120 postnatal cases with 15q11-q13 duplication have been reported, the number of cases with prenatal diagnosis of 15q11-q13 duplication has been less than 20. The clinical manifestations of fetuses with 15q11-q13 duplication during pregnancy, as well as the available screening methods, have not yet been fully elucidated. Expanded noninvasive prenatal screening (NIPS plus) utilizes next-generation sequencing (NGS) for cell-free fetal DNA (cffDNA) and demonstrates excellent performance in detecting fetal copy number variations (CNVs). In this study, the positive predictive value (PPV) of NIPS-plus to detect 15q11-q13 duplications was 100% (7/7). Herein, we report on ten fetuses with 15q11-q13 duplication, review five previously reported prenatal cases in the literature, preliminarily investigate the etiology of 15q11-q13 duplication, and summarized some useful lessons for clinical physicians.

## 2 Materials and methods

### 2.1 Pregnant women collection

Between December 2020 and December 2024, a total of 10 pregnant women with prenatal diagnosis of 15q11-q13 duplication referred to Yiwu Maternal and Child Health Care Hospital and Quzhou Maternal and Child Health Care Hospital for genetic counselling. These women all underwent amniocentesis (at 16–24 weeks of gestation). The average gestational age of the pregnant women were 20 ± 2 weeks. We reviewed their prenatal diagnosis results, relevant genetic testing results, medical records, prenatal manifestations and pregnancy outcomes. The study was approved by the Medical Ethics Committee of Quzhou Maternal and Child Health Care Hospital (approval KY-2023-11), and written informed consent was obtained from the pregnant women.

### 2.2 Prenatal diagnosis

The indications for prenatal diagnosis were as follows: high risk of NIPS plus, abnormal ultrasound, previous fetus/child with abnormalities (Table 1). Amniocentesis was performed using standard approaches under the supervision of experienced providers at each institution. CNVs analysis (using chromosomal microarray analysis or copy number variation sequencing) and karyotype analysis were performed using standard procedures on the amniotic fluid samples. The genomic GRCh37 coordinates of CNVs were lifted over to the genomic GRCh38 coordinates.

**Table 1:** Clinical findings in ten fetuses with 15q11-q13 duplication.

Case	Indications for prenatal diagnosis	Karyotype	The CNVs results	Parental origin	Family history	Pregnancy outcomes
1	High risk of NIPS-plus	Normal	seq[GRCh38]dup(15)(q11.2-q13.1) chr15:g.23374853_28194854dup inherited from the pregnant woman	Maternal origin	The pregnant woman and her mother were carriers of 15q11-q13 duplication and were phenotypically normal.	Termination of pregnancy
2	High risk of NIPS-plus	Normal	arr[GRCh38] 15q11.2q13.1(23123715_28289312) × 3 unknown inheritance	NA	The pregnant woman reported that she could walk at 19 months old and speak at 2 years old. Currently, her intelligence is normal.	After birth, the infant was able to turn over at 6 months old, sit at 9 months old, crawl at 10 months old, walk at 13 months old, and speak at 1.5 years old
3	High risk of NIPS-plus	Normal	arr[GRCh38] 15q11.2q13.1(23123715_28315518) × 3 inherited from the pregnant woman	Maternal origin	The pregnant woman was a carrier of 15q11-q13 duplication and was phenotypically normal.	Termination of pregnancy
4	History of prior adverse pregnancy outcomes	Normal	arr[GRCh38] 15q11.2q13.3(23123715_31118640) × 3 caused by Paternal SV	NA	The pregnant woman and her husband were phenotypically normal.	Termination of pregnancy
5	High risk of NIPS-plus	Normal	arr[GRCh38] 15q11.2q13.1(23387531_28302264) × 3 inherited from the pregnant woman	Maternal origin	The pregnant woman and her husband were phenotypically normal.	Born, but lost to follow-up
6	fetal echocardiography showed ventricular septal defect	Normal	arr[GRCh38] 15q11.2q13.1(23123715_28281759) × 3 inherited from the fetus’ father	Paternal origin	The pregnant woman and her husband were phenotypically normal.	At the 6-month follow-up after birth, the infant was phenotypically normal.
7	High risk of NIPS-plus	47, XN,+psu idic(15)(q13.3)	arr[GRCh38] 15q11.2q13.3(22582283_32623522) × 4 De novo	NA	The pregnant woman and her husband were phenotypically normal.	Termination of pregnancy
8	High risk of NIPS-plus	Normal	arr[GRCh38] 15q11.2q13.1(23123715_28281759) × 3 inherited from the pregnant woman	Maternal origin	The pregnant woman, her sister, and her mother were all carriers of 15q11-q13 duplication and were phenotypically normal.	Born, but lost to follow-up
9	Advanced maternal age	Normal	arr[GRCh38] 15q11.2q13.1(22582283_28769879) × 3 De novo	Maternal origin	The pregnant woman and her husband were phenotypically normal.	Termination of pregnancy
10	High risk of NIPS-plus	Normal	arr[GRCh38] l15q11.2q13.1(23455960_28294829) × 3 De novo	Paternal origin	The pregnant woman and her husband were phenotypically normal.	Termination of pregnancy

The CNVs results were based on the genomic GRCh38 coordinates.

NA, not available; SV, structure variation.

### 2.3 Parental origin and inheritance of the duplication

To investigate the parental origin and inheritance of the duplication, the fetuses’ parents were recommended to perform karyotype analysis, CNVs analysis, multiplex ligation-dependent probe amplification (MLPA), or fluorescence *in situ* hybridization (FISH) on peripheral blood. None of these methods were mandatory, but all parents selected at least one method. Written informed consent was obtained from the parents.

### 2.4 Genetic counselling and pregnancy outcomes

All pregnant women who received prenatal diagnosic reports were provided with comprehensive genetic counseling and fully informed of the relevant risks. Under the premise of complying with national policies, all pregnant women independently made decisions regarding the outcome of their pregnancies. Follow-up was conducted via telephone to ascertain pregnancy outcomes, diagnoses, and postnatal care measures.

### 2.5 Literature review

We searched the PubMed database using “15q11-q13 duplication” and “fetal/fetus” as keywords. The search period covered from the establishment of the databases to January 31, 2025. We reviewed prenatal cases with availability of clinical data, including relevant genetic testing results, prenatal manifestations and pregnancy outcomes. A total of four prenatal cases with three copies of 15q11-q13 duplication and one case with four copies of 15q11-q13 duplication were recorded in three literature (He et al., 2023; Song et al., 2022; Kang et al., 2021).

## 3 Results

We retrospectively reviewed a total of 10 fetuses with 15q11-q13 duplication (Table 1). 90% (9/10) of fetuses with 15q11-q13 duplication did not show abnormal manifestations, except for one fetus that had the ventricular septal defect (VSD). Herein, the positive predictive value (PPV) of NIPS-plus to detect 15q11-q13 duplications was 100% (7/7). Three 15q11-q13 duplications were *de novo* (Case 7 was not tested for the parental origin of the dup15q, Case 9 and 10 was tested), four 15q11-q13 duplications were inherited from the pregnant women, one 15q11-q13 duplication was inherited from the fetus’s father, one 15q11-q13 duplication was caused by paternal genomic structure variation (SV), the inheritance of the duplication in case 2 was unknown. The analysis of parental origin in seven fetuses revealed that 71.4% (5/7) of the 15q11-q13 duplications were maternally derived. 60% (6/10) of pregnant women eventually chose to terminate their pregnancies.

## 4 Discussion

The chromosome 15q proximal region contains five low copy repeats (LCRs) regions that harbor five known breakpoints (BP1-5) and mediate recurrent genomic rearrangements (such as deletions, duplication, inversions) through non-allelic homologous recombination (NAHR) (Burssed et al., 2022). The well-characterized Prader Willi syndrome (PWS)/Angelman syndrome (AS) critical region (PWACR) is located between BP2 and BP3 within chromosome 15q11.2-q13.1 (Ma et al., 2023). Although PWS and AS are both associated with abnormalities in the same region of chromosome 15q11-q13, the main difference in their pathogenic mechanisms lies in the different parental origin of the abnormal 15q11-q13 region (excluding the copy number gain in this region), which ultimately leads to different manifestations (Louveau et al., 2023). 15q11-q13 microduplication syndrome (MONDO:0012081) is defined as the presence of three or more copies of 15q11.2-q13.1 that must contain the PWACR region (genomic GRCh38 coordinates chr15:22,782,170-28,134,72, ClinGen ID: ISCA-37404).

Due to the difference between paternally and maternally expressed PWACR, the maternally or paternally derived duplication of 15q11.2-q13.1 presents with various clinical manifestations. The maternal 15q11-q13 duplication syndrome is characterized by developmental delay, intellectual disability, autism spectrum disorder (ASD), seizures, and dysmorphic features (Beghi et al., 2020). The gene associated with Angelman syndrome, *UBE3A,* located on 15q11.2, is maternally expressed in postnatal neurons. Neuronal overexpression of *Ube3a* in mice exhibited anxiety-like behaviors, learning impairments, and reduced seizure thresholds (Copping et al., 2017). The *HERC2* gene, located on 15q13.1, encodes a 527 kDa E3 ubiquitin ligase involved in cell cycle regulation, neuronal development, mitochondrial function, and DNA repair, and is associated with neurodevelopmental disorders (Elpidorou et al., 2021). It has been found to exhibit increased expression in the neurons of individuals with maternal 15q11-q13 duplication (Urraca et al., 2018).

The paternally derived 15q11-q13 duplication has been considered a low-penetrance CNV. A few cases with paternally derived duplication have been reported, presenting with normal or milder phenotypes, such as ASD, mild learning difficulties, intellectual disability, seizures, and dyslexia (Marini et al., 2013). One study found that a paternally expressed gene, *Necdin* (*Ndn*), was as a driver gene for paternal 15q11q13 duplication, resulting in the development of ASD-like phenotypes in mice (Tamada et al., 2021).

Herein, we report 10 fetuses with prenatal diagnosis of 15q11-q13 duplication. Some findings from these cases are documented as follows:

1. NIPS-plus is a cost-effective screening tool for 15q11-q13 duplication.

NIPS plus is an efficient and cost-effective screening approach that is highly recommended. In this cohort, the PPV of NIPS plus to detect 15q11-q13 duplication was 100% (7/7), which was in accordance with another study (Parijs et al., 2024). Most of the fetuses (90%) with 15q11-q13 duplication did not show ultrasound abnormalities in this cohort. Although six prenatal cases have been reported (Bisba et al., 2024), these cases were excluded from the review due to the unavailability of clinical data. The referral reasons of the five recorded prenatal cases were ultrasound abnormalities (one fetus was diagnosed with Tetralogy of Fallot), advanced maternal age (two fetuses), high risk of NIPS plus (one fetus), and a positive Down’s syndrome screening test (one fetus) (Supplementary Table 1). Two independent studies (He et al., 2023; Song et al., 2022) have each reported a fetus with maternally derived 15q11-q13 duplication. Coincidentally, the ultrasound examinations of these two unrelated fetuses both revealed intrauterine growth restriction (IUGR) at 26 weeks of gestation after amniocentesis. Therefore, as we can see, without the recommendation for NIPS screening or amniocentesis, most of fetuses and pregnant women would not have been aware that they were carriers of the 15q11-q13 duplication.

1. The penetrance of maternally derived 15q11-q13 duplication incomplete.

The one extra maternal copy of 15q11.2-q13.1 appeared to show incomplete penetrance, some individuals with maternal 15q11.2-q13.1 duplication appeared unaffected or exhibited only slight features. In two pedigrees with maternal 15q11.2-q13.1 duplication, the pregnant women (Case 1, Case 8), the mothers of the pregnant women (Case 1, Case 8), and the sister of the pregnant woman (Case 8) were all carriers of the 15q11.2-q13.1 duplication and were phenotypically normal. To date, more than 120 phenotypically abnormal cases with maternal 15q11-q13 duplication have been reported, but few studies have reported phenotypically normal cases with maternal 15q11.2-q13.1 duplication. Isles et al. (2016) reported four phenotypically normal individuals with maternal 15q11.2-q13.1 duplication. They estimated the penetrance of maternal 15q11.2-q13.1 duplication for developmental delay/multiple congenital anomalies/ASD to be 50.5%, which was approximately 2.5 times higher than that of paternal origin (20.7%). There are also viewpoints considering that the penetrance of an extra maternal copy of 15q11.2-q13.1 is complete and variable expressivity, and that milder or slight clinical manifestations are often easily overlooked (Han et al., 2021).

1. Genomic structural variations could cause 15q11-q13 duplication.

**Figure 1:**
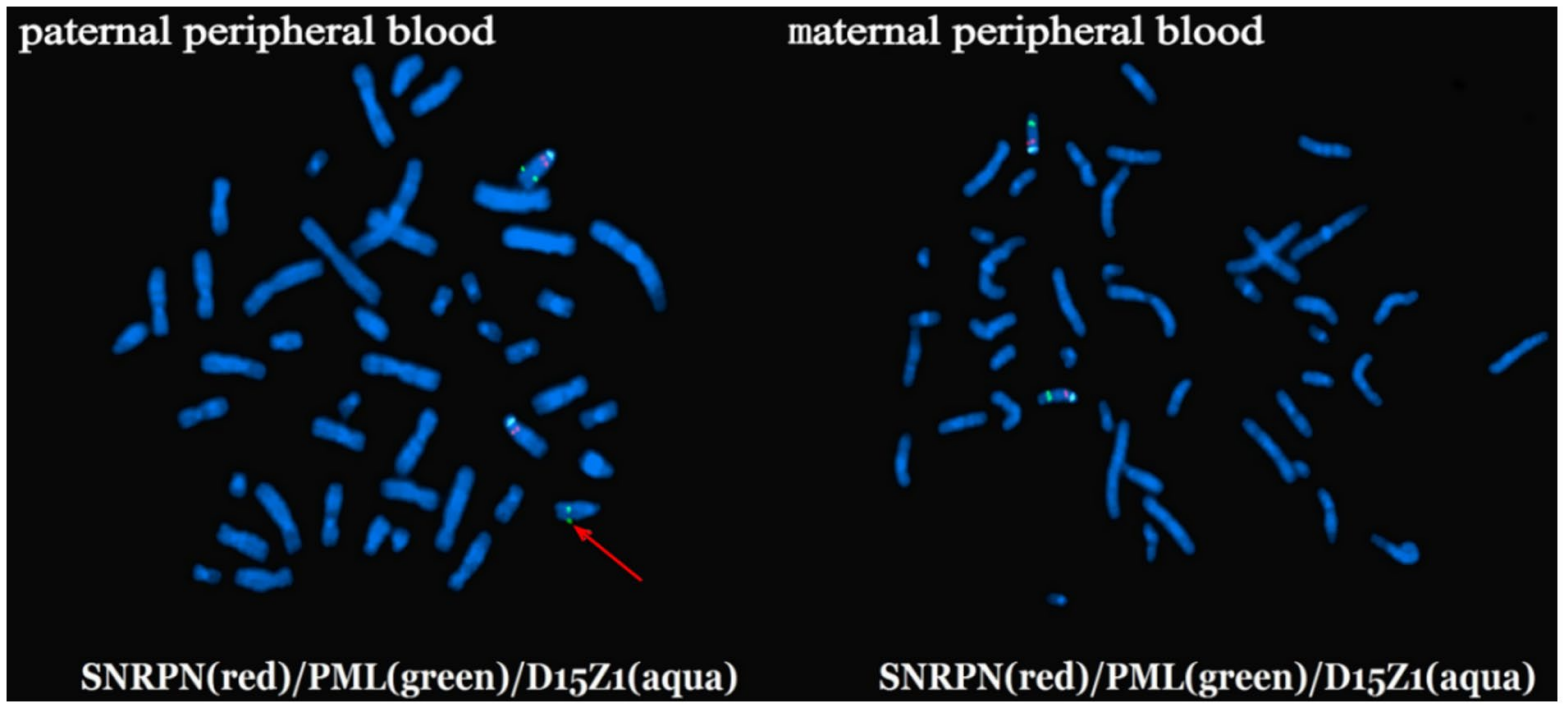
The FISH results of parents’ peripheral blood. FISH analysis of the paternal peripheral blood using probe D15Z1 (spectrum aqua) at 15p11.2, SNRPN (spectrum red) at 15q12, and PML (spectrum green) at 15q24.1 showed a chromosomal translocation involving the 15q24.1 and the end of chromosome 14q (marked by the red arrow). FISH result of the maternal peripheral blood showed normal.

Not all 15q11-q13 duplications were attributed to the parental inheritance, investigation the etiology of 15q11-q13 duplication necessitates the application of multiple molecular technologies. We reported a novel case (Case 4) with 15q11-q13 duplication caused by a paternal cryptic chromosomal translocation. The 34-year-old pregnant woman (gravida 3, para 1) underwent amniocentesis due to a history of prior adverse pregnancy outcomes (one stillborn fetus, one phenotypically abnormal child with 15q11-q13 duplication, one fetus with 15q11-q13 deletion). FISH analysis was performed for parents using specific probes (Vysis) according to the manufacturer’s instructions. FISH analysis of the paternal peripheral blood using probe D15Z1 (spectrum aqua) at 15p11.2, SNRPN (spectrum red) at 15q12, and PML (spectrum green) at 15q24.1 preliminarily indicated the presence of a cryptic chromosomal translocation between chromosome 15q24.1 and the end of chromosome 14q (Figure 1). We hypothesized that the paternal chromosomal translocation resulted from NAHR mediated by interchromosomal LCRs between chromosome 15q and chromosome 14q. Although such cases are rarely reported, Vieira et al. (2024) have reported one child with 15q11-q13 duplication syndrome caused by a *de novo* maternal cryptic balanced double translocation involving chromosomes 13, 19, and 15. Therefore, in cases where it is clinically indicated, particularly when there is a history of prior adverse pregnancy outcomes, the parents’ genomic structural variations should be evaluated.

1. Pregnant women tended to terminate their pregnancies in China when a chromosomal abnormality was detected.

Regardless of whether the 15q11-q13 duplication was maternally or paternally derived, a majority of parents (60%, 6/10) in this Chinese cohort opted to terminate the pregnancy due to concerns about potential risks. Due to China’s unique religious beliefs and national policies, attitudes towards terminating pregnancies may differ from those in other countries. To date, there is a lack of comparable reports on pregnancies terminated due to 15q11-q13 duplication in the existing literature. Recently, a study from China reported that the termination of pregnancy (TOP) rate for low-penetrance pathogenic CNVs (with penetrance less than 30%) was 40.9% (18/44) (Liu et al., 2025).

In conclusion, most fetuses with 15q11-q13 duplications did not have ultrasound abnormalities, but they may have congenital heart disease. NIPS plus is a reliable and effective method for the early detection of fetuses with 15q11-q13 duplication. The penetrance of maternal derived 15q11-q13 duplication is incomplete. It is recommended to perform parent-of-origin testing to assist in the investigation of the etiology of 15q11-q13 duplication and decision-making regarding pregnancy outcomes. Given that the rate of termination of pregnancy for 15q11-q13 duplications in China seems relatively high, genetic counseling needs to be carried out with caution.

## Data Availability

The original contributions presented in the study are included in the article/Supplementary material, further inquiries can be directed to the corresponding author.
