# Supplementary material for: Case Report: Prenatal diagnosis of 10 fetuses with 15q11-q13 duplication and pregnancy outcome in a cohort of Chinese women
Source: Front Neurosci. 2025 Sep 18;19:1613797. doi: 10.3389/fnins.2025.1613797 (PMC12488580; doi:10.3389/fnins.2025.1613797)
Supplement: Supplementary file 1 [file Table_1.DOCX]

| **Supplemental table 1:** Clinical information with prenatal diagnosis of 15q11-q13 duplication in literature | | | | | | |
| --- | --- | --- | --- | --- | --- | --- |
| literature | Indications for prenatal diagnosis | Karyotype | The CNVs result of prenatal diagnosis | Parental origin | Family history | Pregnancy outcomes |
| Kang et al (2021)[^[[1]](#endnote-0)^] | Tetralogy of Fallot | NA | arr[GRCh37] 15q11.1-q13.3  (20686219_3289952)×3  **unknown** inheritance | NA | NA | Termination of pregnancy |
| Song et al (2022)[^[[2]](#endnote-1)^] | High risk of Down’s syndrome screening test | Normal | arr[GRCh37]  15q11.2q13.1(23290789_28928729)×3  **inherited** from the fetus’ father | Paternal origin | The pregnant woman and her husband were phenotypically normal. | At 39 weeks of gestation, the mother gave birth vaginally to a  male baby weighing 3550 g. At 36-month  checkup, the baby’s development was normal. |
|  | Advanced maternal age | Normal | arr[GRCh37]  15q11.2q13.1(23894551_28107155)×3  **inherited** from the pregnant woman | Maternal origin | The pregnant woman and her husband were phenotypically normal. | At 26 weeks of gestation, ultrasound examination showed IUGR in the fetus. The parents decided to terminate the pregnancy. |
|  | High risk of NIPS-plus | 46,XX,+mar | arr[GRCh37] 15q11.2q13.3(22770420_32439250)×4  **De novo** | Maternal origin | The pregnant woman and her husband were phenotypically normal. | Termination of pregnancy at 24 weeks of gestation. |
| He et al (2023)^[^[[3]](#endnote-2)^]^ | Advanced maternal age | Normal | arr[GRCh37]  15q11.2q13.1(23894550_28107154)×3  **inherited** from the pregnant woman | Maternal origin | **The pregnant woman, the pregnant woman’s sister and mother were all** carriers of 15q11-q13 duplication and were phenotypically **normal**. | At 26 weeks of gestation, an ultrasound examination revealed IUGR in the fetus. The parents decided to terminate the pregnancy. |
| NA: not available, IUGR: intrauterine growth restriction | | | | | | |

1. [] Kang J, Lee CN, Su YN, et al. The Prenatal Diagnosis and Clinical Outcomes of Fetuses With 15q11.2 Copy Number Variants: A Case Series of 36 Patients. Front Med (Lausanne). 2021;8:754521. doi:10.3389/fmed.2021.754521 [↑](#endnote-ref-0)
2. [] Song J, Liu X, Zhang C, Xu F, Wang B. Clinical and genetic study of three families with 15q11q13 duplications. Taiwan J Obstet Gynecol. 2022;61(4):717-721. doi:10.1016/j.tjog.2021.12.004 [↑](#endnote-ref-1)
3. [] He L, He C, Tang W. Prenatal Diagnosis and Genetic Counseling of a Maternally Inherited Chromosome 15q11.2q13.1 Duplication in a Chinese Family. Altern Ther Health Med. 2023;29(7):462-464. [↑](#endnote-ref-2)
